# Silane-Coupled Silica Nanoparticles Encapsulating Emitting Quantum Dots: Advancing Robust Phosphors for Displays and Beyond

**DOI:** 10.3390/molecules30163369

**Published:** 2025-08-13

**Authors:** Norio Murase, Chunliang Li

**Affiliations:** 1 Kansai Collaboration Center, National Institute of Advanced Industrial Science and Technology (AIST), Ikeda 563-8577, Osaka, Japan; 2 Quantum Materials Technology Co., Ltd. (QMT), 2-22-11 Obana, Kawanishi 666-0015, Hyogo, Japan; 3 School of Materials Science and Engineering, Tianjin University of Technology, Xiqing, Tianjin 300384, China

**Keywords:** sol-gel, silane-coupling agent, colloidal quantum dot, photoluminescence, protection

## Abstract

Colloidal quantum dots (QDs) are semiconductor crystals a few nanometers in size. Due to their vibrant colors and unique photoluminescence (PL), QDs are widely utilized in displays, where barrier films provide essential shielding. However, one of the primary challenges of QD applications remains achieving sufficient robustness while keeping costs low. Over the past two decades, significant progress has been made in the encapsulation of QDs within silica matrices, aiming to preserve their original PL properties. Research efforts have evolved from bulk forms to thin films. Silica nanoparticles containing multiple embedded QDs have emerged as particularly promising candidates for practical applications. This review highlights recent advancements in silica-based QD encapsulation, incorporating findings from both the authors’ investigations and those of other research groups within the field. Silica glass possesses inherent shielding capabilities, but silane coupling agents such as (3-aminopropyl)trimethoxysilane and (3-mercaptopropyl)trimethoxysilane tend to negatively impact this functionality when they are used alone, partly because of the limited formation of a well-developed glass network structure. However, when judiciously controlled, they can serve as mediators between the QD surface and the surrounding pure silica glass matrix, helping to preserve PL properties and control the morphology of silica particles. This review discusses the potential for achieving exceptional shielding properties through sol–gel glass fabrication at low temperatures, utilizing both tetraethoxysilane and other silane coupling agents.

## 1. Introduction

Silica, particularly in its amorphous form (silica glass), plays a crucial role in the semiconductor industry because of its exceptional protective, insulating properties and adaptability in shaping [1]. This distinctive nature may also be indispensable as a matrix for quantum dot (QDs) emissions. Throughout this article, amorphous silica or silica glass is collectively referred to as “silica”. Since the QDs discussed here are synthesized in solution (colloidal QDs), the silica is similarly prepared in solution via the sol–gel method to ensure compatibility. In contrast to the vacuum-based processes commonly employed in the semiconductor industry, the solution-phase chemical reactions required to form protective layers on QDs are dependent on the use of silane coupling agents. This contribution to the Special Issue on hybrid organic/inorganic (O/I) sol–gel-derived nanocomposite systems summarizes accumulated knowledge on the preparation and evaluation of sol–gel-derived silica-encapsulated QD emissions. Relevant studies from other research groups are also critically examined to provide a comprehensive overview.

It has been more than a decade since the first commercial release of televisions utilizing QDs as photoluminescence (PL) sources, commonly referred to as QD TVs [2]. The primary application of QDs continues to be as phosphors in display technology, with the overall market size reportedly valued at billions of dollars annually [3]. Another potential use is in biomedical tagging, where QDs function as fluorescent reagents [4,5,6]. However, both applications still demand enhanced robustness in QD formulations. Following this introductory section, Section 2 presents a concise overview of the related research areas, with emphasis on colloidal QDs and their protective materials, and a brief outline of the structure and scope of this article. This discussion aims to facilitate a better understanding of subsequent arguments. Section 3 outlines the basic principles of the preparation of silica matrices with encapsulated emitting QDs, along with the results observed in bulk and thin-film forms. Given their promising potential for applications, silica nanoparticles with encapsulated QDs are particularly noteworthy. Accordingly, the subsequent Section 4 explores four distinct nanoparticle preparation methods, categorized based on two types of QDs (hydrophilic and hydrophobic), and two sol–gel approaches (reverse micelle and the Stöber method). Related research from other groups is also provided. Among these, the preparation of silica nanoparticles with dozens of encapsulated hydrophobic QDs using the Stöber method yielded the most promising results, with the aid of a silane coupling agent. As this particular method demonstrated significant advantages, Section 5 delves deeper into preparations and evaluations of these silica particles, despite their classification under hydrophobic QD encapsulation by the Stöber method in Section 4. Recent advancements, applications, and challenges in the field are discussed in Section 6. Finally, Section 7 offers concluding remarks with perspectives on future developments. Table 2, discussed later, is intended to facilitate understanding of the positioning and classification outlined in this review.

## 2. Background of Related Fields and Article Outline

### 2.1. Colloidal Quantum Dots

**Figure 1:**
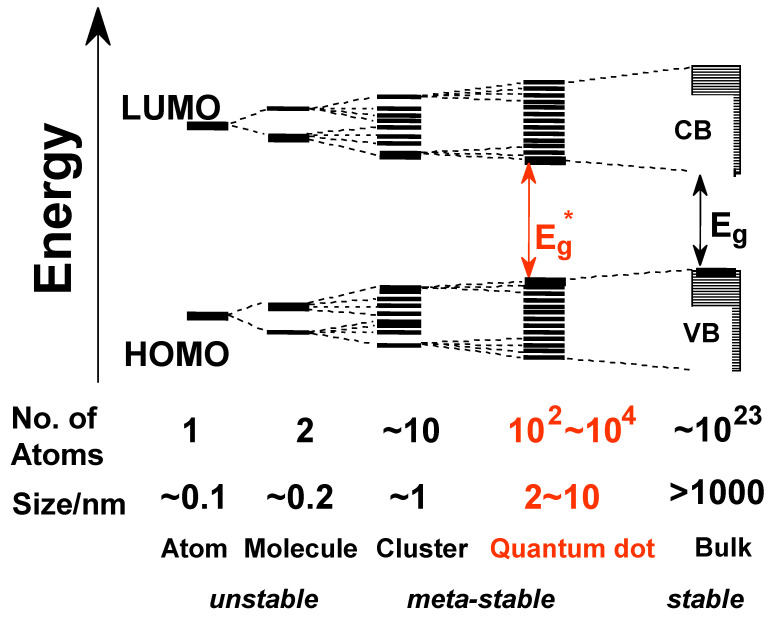
The relationship between particle size and band gap in semiconductors. As particle size decreases, quantum confinement effects become more pronounced, leading to an increase in band gap energy. This phenomenon plays a crucial role in tuning optical and electronic properties in nanoscale semiconductor materials, such as QDs.

**Figure 2:**
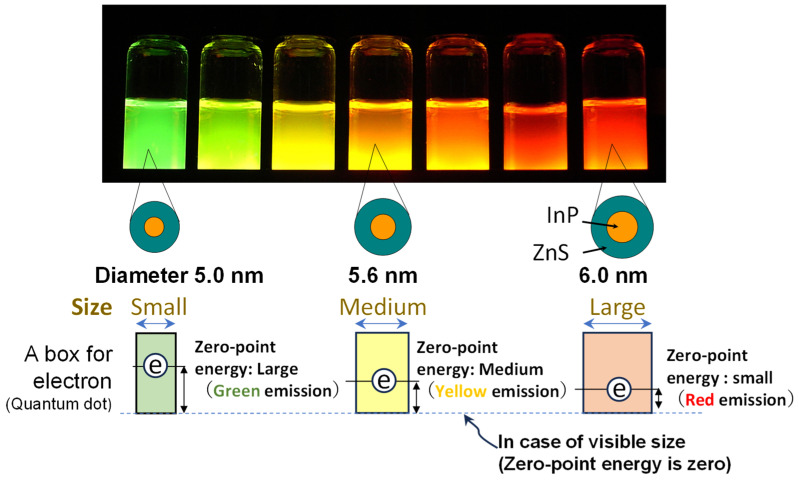
Schematic representation of the quantum size effect. As the QD size decreases, quantum confinement effects become more pronounced, leading to a widening of the bandgap, according to the value of zero-point energy originating from the uncertainty principle [7] (Courtesy of AIST, Japan). This effect was first observed and precisely explained by two Nobel laureates in chemistry: Dr. A. I. Ekimov [8], and Dr. L. E. Brus [9].

**Figure 3:**
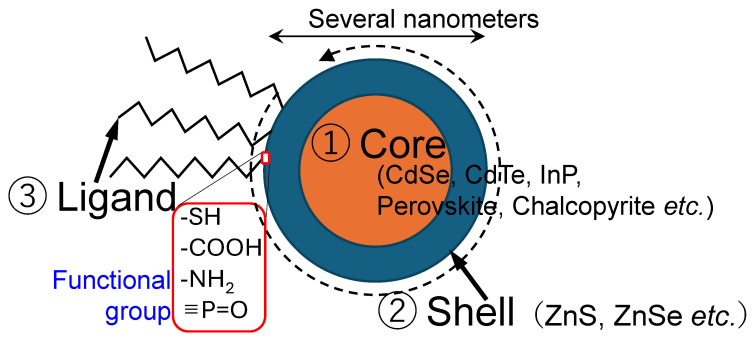
Schematic representation of the structure of emitting QDs, consisting of three primary components: ① the core, which defines the fundamental optical and electronic properties; ② the shell, which enhances stability and emission efficiency; and ③ the surrounding ligand molecules, which facilitate dispersion and surface interaction. The functional group of PO in the figure is from trioctylphosphine oxide. In certain cases, the shell is embedded within the core structure during synthesis, as exemplified by aqueous CdTe QDs [10]. Perovskite QDs exhibit strong PL originating predominantly from their core structure; however, the presence of an outer shell significantly improves their structural robustness and stability [11].

Colloidal QDs are semiconductor nanocrystals synthesized in solution, typically measuring a few nanometers in diameter. Their size places them between atomic clusters and bulk materials, as illustrated in Figure 1, with each QD crystal consisting of approximately 10^2^–10^4^ atoms per particle. The quantum size, also known as quantum confinement, causes the band gap to widen as the crystal size decreases, actually originating from the uncertainty principle, which is schematically depicted in Figure 2 [7]. Emitting QDs generally consist of three components: a core, a shell, and surface ligands (surfactants), as shown in Figure 3. To achieve narrow and intense PL, the band gap of the shell must be larger than that of the core. The ligand structure also influences the solubility properties of QDs. Hydrophilic QDs are typically functionalized with ligands containing short carbon chains (fewer than three–four carbon atoms). Conversely, hydrophobic QDs are stabilized during synthesis using ligands with longer carbon chains (generally exceeding seven carbon atoms). Both hydrophilic and hydrophobic colloidal QDs are precipitated upon the addition of lower alcohols such as ethanol and methanol. This purification method is generally used during synthesis. Following this process, ligand exchange is frequently performed post-synthesis to tailor surface properties for specific downstream applications. Given their small size, a large proportion of QD atoms are surface-exposed, making surface chemistry a critical factor in determining PL efficiency.

The most typical hydrophilic emitting QD widely studied is CdTe, capped with low molecular weight thiols such as thioglycolic acid [12]. In the case of hydrophobic QDs, the most investigated and highest-quality QDs are those that are CdSe-based, pioneered by the success of hot injection synthesis [13]. Their shells, typically composed of CdS, ZnSe, or ZnS, enable a remarkably narrow PL spectral width (approximately 25 nm or less in full width at half maximum, FWHM), ensuring exceptional color fidelity in display applications. However, cadmium-based QDs are being phased out due to RoHS restrictions [14]. As an alternative, InP-based QDs currently stand out as the sole viable, environmentally friendly binary QDs capable of emitting visible light through quantum size effects [15]. Despite early awareness of their potential, research on InP QDs lagged behind that of CdSe by more than a decade, largely due to the challenging reaction conditions inherent to III–V semiconductor systems and the difficulty of forming an optimized interface between InP cores and II–VI shells (such as ZnSe or ZnS). After multiple trials [16,17,18], InP QDs finally achieved nearly 100% PL quantum yield (PLQY) [19,20]. Currently, high-end displays have begun adopting InP-based QDs as an alternative to CdSe-based QDs, despite their relatively broad PL spectral width (typically exceeding 35 nm FWHM).

In addition to traditional binary QDs (II–VI and III–V), two-types of ternary QDs, namely chalcopyrite (I-III-VI_2_ or II-IV-V_2_, such as CuInS_2_, AgInS_2_, and sometimes quaternary) and perovskite (CH_3_NH_3_PbX_3_ or CsPbX_3_, where X = Cl, Br, I), have emerged. These materials show narrow and intense PL (typically less than 25 nm), comparable to CdSe-based QDs.

Non-toxic chalcopyrite QDs were first reported as Zn-CuInS_2_ in 2006 [21]. These QDs exhibited PL accompanied with strong defect emissions, which likely originated from deviations in stoichiometric composition due to the limited number of atoms within individual QDs. In the case of AuInS_2_ QDs, the formation of a specific shell structure significantly reduced the defect emission [22]. However, the suppression was not entirely complete. Ongoing efforts continue to improve the PL properties and processability of these QDs [23]. Several studies have also reported the silica encapsulation of these QDs to enhance their stability, as briefly outlined in Section 2.4.

Lead-based perovskites represent a distinct class of emitting QDs. Although their synthesis procedures are broadly similar to those used for traditional hydrophobic II-VI, they are highly ionic. Unlike other QDs, their emission is largely insensitive to the surface conditions [24], although the shell formation is effective for structural protection [11]. Currently, stability remains a significant drawback for these QDs as well. Consequently, extensive research has been conducted on their encapsulation within silica matrices, as outlined in Section 2.3. Lead (Pb), a regulated heavy metal, presents environmental and safety concerns in consumer applications. However, ongoing advancements in solar cell technologies, employing the same perovskite compositions in bulk rather than QD form, have contributed to a relaxation of regulatory constraints for QD-based consumer products.

The remaining class consists of single-component (unary, group IV) QDs. Carbon-based QDs are quite sensitive to the surface, and their PL is typically characterized by a broad emission profile [25]. Similarly, silicon QDs generally show broad PL spectra, and both systems continue to face significant challenges in terms of stability [26]. To date, no substantial studies have been identified regarding the incorporation of these unary QDs into silica matrices.

### 2.2. Matrices for QD Encapsulation

As discussed above, QDs require coating and protection with another transparent material to preserve their distinctive PL properties. Table 1 summarizes research efforts dedicated to the shielding of emitting QDs, including contributions from key producers [27,28,29,30], the authors’ own investigations, and various polymer-based approaches. Attempts to encapsulate QDs within crystalline materials have largely been unsuccessful. Since QDs themselves are crystals, achieving a stable interface with another crystalline matrix is challenging, particularly at high dispersion concentrations. This often leads to surface defects that reduce PLQY. Among transparent amorphous materials, polymer and glass (such as silica glass) stand out as viable encapsulation matrices. Due to the significantly lower oxygen permeability of glass compared to polymers [31], glass is widely regarded as the optimal choice for QD encapsulation. Consequently, thousands of publications have explored sol–gel-derived silica as a protective encapsulation medium. Representative examples will be discussed following categorical classification later in this work. However, due to the complexity of sol–gel processing and the relatively lower performance of the resulting materials, most studies in this area have not been actively pursued. Nonetheless, the continued and substantial contributions made by the authors’ research group offer a valuable foundation for interpreting progress in this field. Accordingly, the authors encourage readers to consider these findings as a core framework of this review for understanding the development and future prospects of silica-encapsulated QD technologies, as elaborated in the following subsection.

### 2.3. Outline of the Article

Table 2 summarizes the methods for the encapsulation of emitting colloidal QDs in silica matrices, which are categorized from letters A to F, corresponding to the respective sections discussed in this article.

Briefly, two types of hydrophilic and hydrophobic QDs, immediately after synthesis, were encapsulated using the sol–gel process of reverse micelle or the Stöber method, and other approaches. Given that current display technologies primarily employ binary QDs based on CdSe and InP (with ZnSe-based variants used in limited demonstration stages), research activities have largely concentrated on these materials. The results of authors’ studies are compared with existing reports, and it is concluded that F, corresponding to the encapsulation of hydrophobic QDs into silica particles assisted by silane coupling agents, yields the most favorable outcomes.

### 2.4. Silica Encapsulation of Ternary QDs

A selection of representative studies on the silica encapsulation of ternary QDs is discussed in this subsection. The results are interpreted with reference to the encapsulation categories defined in Table 2.

Chalcopyrite QDs (CuInS_2_-based) have been encapsulated within silica nanoparticles using tetraethoxysilane (TEOS) via reverse micelle methods. Both hydrophilic (E) [32] and hydrophobic (A) [33] variants have been investigated, though only the latter was reported to have a PLQY of 1.5%. Stability assessments indicated limited improvement upon encapsulation when compared to the pre-encapsulated state. Additionally, silane coupling agents, including (3-aminopropyl)trimethoxysilane (APS), have been employed for CuInS_2_ QDs (hydrophobic version of No. B) [34,35,36]. The silica precursor used in reference [34] was not a conventional silane coupling agent, but it exhibited reaction behavior closely resembling such agents. Overall, PLQYs achieved using this approach are generally superior to those obtained via the reverse micelle method. However, the degree of glass network formation appears suboptimal, as indicated by the absence of noticeable shrinkage in the resulting monoliths upon completion of the preparation process. The stability of silica-encapsulated chalcopyrite QDs exceeds that of their pristine counterparts [35]. For AgInS_2_-based QDs, only one instance of successful silica encapsulation has been identified, involving growth within a molten glass matrix. This approach yielded broad PL emission across the visible spectrum [37].

Perovskite QDs have been more actively integrated into silica matrices compared to other QD systems. The predominant approach involves standard sol–gel methods utilizing silane coupling agents such as APS [38,39,40] or alkoxides with four functional groups such as TEOS [41] or tetramethoxysilane (TMOS) [42,43], while the latter exhibits a significantly higher hydrolysis rate than that of TEOS. These encapsulation strategies are classified under B, targeting hydrophobic QDs. A maximum PLQY of 78% was reported for QDs encapsulated using APS [38]. However, stability assessments in these studies were limited to comparisons with pristine QDs or those embedded in polymethyl methacrylate (PMMA) [38]. In another work, hydrophobic QDs were encapsulated using a modified Stöber method (F), with stability evaluated relative to their pristine counterparts [44].

Commercial mesoporous silica matrices have been employed to incorporate colloidal QDs [45,46,47]. However, no comparative data on stability with other solid matrices has been reported. Among the available studies, the most promising one appears to be the synthesis of CsPbBr_3_ QDs within a molten glass matrix, achieving a PLQY of 42% [48]. Although the size distribution of QDs in the glass is broad, these QDs are stable in air for over one month. A comprehensive investigation has described the growth mechanism of these QDs in glass matrices at elevated temperatures, such as 600 °C, over extended durations exceeding 10 h. The study combines experimental results with theoretical insights into particle growth mechanisms [49]. Nevertheless, a significant limitation of this method is the inability to purify and further modify the QDs post-incorporation, such as enhancing PLQY after synthesis [50]. Therefore, the authors have advocated for research into dispersing colloidal QDs into silica matrices through sol–gel approaches following colloidal synthesis.

Here, it is worth noting that most of the encapsulation methods for ternary QDs emerged in the 2010s. These early research attempts failed to result in systematic or quantitative studies, limiting their impact on methodical development and comparative analysis.

**Table 2:** Overview of binary QD emission incorporated into silica glass matrices using various sol–gel methods. The encapsulation approaches are categorized from A to F, corresponding to detailed discussions provided in relevant sections of this article. The non-English term (nano glass in Japanese) in the image of category No. B is the name of research project.

Category No.	Quantum Dot	Interaction with Water	Silane-Coupling Agent	Method	Morphology of Silica Glass	Comments	This Article	Main Year	References
**A**	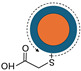 CdTe, ZnSe, & InP-based	Hydrophilic	—	Reverse micelle	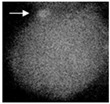 Nanoparticle	Not incorporated into glass spheres.	Section 4.1	2004	[51,52,53]
**B**			APS	(Normal sol-gel)	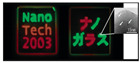 Bulk	First successful incorporation into glass with high PLQY; however, the glass dissolved upon exposure to hot water.	Section 3.1	2004	[54,55,56,57,58]
**C**			APS	Layer-by-layer	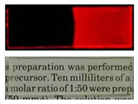 Thin film	High QD loading achieved; however, the resulting film was fragile.	Section 3.2	2005	[59]
**D**			MPS	Stöber	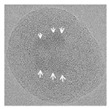 Nanoparticle	Porosity was characterized; QDs exhibited a broad FWHM.	Section 4.2	2010, 2020	[60,61,62,63]
**E**	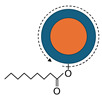 CdSe, & InP-based	Hydrophobic	—	Reverse micelle	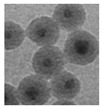 Nanoparticle	Single QD per silica particle achieved; however, dispersion concentration remained insufficient.	Section 4.3	2006	[64,65,66,67,68,69]
**F**			MPS	Modified Stöber	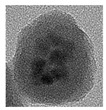 Nanoparticle	Multiple QDs per silica particle; extensively investigated due to several applications.	Section 4.4, and Section 5 and Section 6	2010–2025	[70,71,72,73,74,75,76,77]

## 3. Incorporation of QDs in Bulk and Thin-Film Silica Derived from Silane Coupling Agents

### 3.1. Bulk Incorporation of QDs Using APS

Following the successful synthesis of hydrophobic CdSe-based QDs [78], these QDs were incorporated into polymer matrices molded within glass tubes for industrial applications [79]. However, the reported PLQY remained relatively low, ranging between 20 and 40%. Around the same time, researchers introduced a modified sol–gel method to disperse QDs within silica [54]. However, the prepared matrix is seemingly a gel, not a solid. Unlike polymer-based dispersions, the sol–gel approach requires sufficient reaction time to achieve uniform solidification, a critical factor in optimizing the method.

Building upon the fundamental properties of colloidal emitting QDs, a series of investigations was conducted to evaluate their stability and optimize incorporation into silica matrices. Generally, the radius (*r*) of QDs in solution grows or dissolves according to the equation [80]

(1) $$\frac{dr}{dt}=\frac{K}{r}\left(\frac{1}{r_{c}}-\frac{1}{r}\right),$$

where *K* is a constant determined by the solution temperature, the diffusion coefficients of the components, and the specific surface energy of QDs. When the radius *r* of QD is smaller than a critical value *r*_c_, it dissolves and finally disappears into the solution. The dissolution process leads to a rapid and significant decrease in PLQY. The constituent atoms or clusters of such QDs are used to grow other QDs larger than *r*_c_. This phenomenon is known as Ostwald ripening. To create bulk silica glasses incorporating QDs, aqueous CdTe-based QDs stabilized with thioglycolic acid (TGA) were initially selected. The core ligand structure of TGA features a single carbon skeleton, offering minimal steric hindrance and facilitating uniform dispersion within the sol–gel matrix.

Based on Equation (1), a tailored protocol was developed to preserve the initial PLQY of QD after the incorporation into silica matrices.

Selection of Silane Coupling Agent: The amino-functionalized silane (APS) was chosen to facilitate stable QD dispersion. This selection leveraged the carboxylic acid groups present on TGA-stabilized QDs. The amine functionality not only exhibits affinity toward carboxyl groups facilitating coordination with QD surface ligands, but also serve as a catalyst for gel formation during the sol–gel process [54].

Dispersion Strategy: Constituent molecules and ions including TGA and Cd^2+^ were dispersed in APS solution to minimize the dissolution of QDs during the sol–gel process. In other words, Equation (1) indicates that the dissolution of QDs is effectively suppressed when the particles are surrounded by a sufficient concentration of constituent molecules and ions. This protective environment helps stabilize the QDs by minimizing surface degradation and maintaining their PL properties.

Optimized Timing for QD Addition: To mitigate time-dependent dissolution, QDs were introduced into the sol phase immediately prior to gelation. This timing ensures rapid immobilization within the forming silica network, effectively preventing QD dissolution and preserving their structural integrity and PL properties.

**Figure 4:**
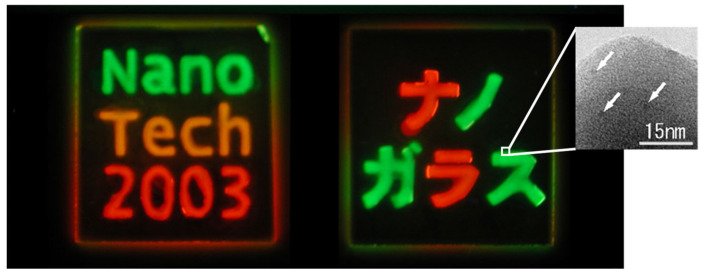
Fluorescent color images of the prepared bulk-type glass containing QDs under UV lamp excitation at 365 nm. The phosphor is selectively adhered to patterned grooves on the surface of the glass substrates, enhancing localized emission [55]. The non-English term (nano glass in Japanese) is the name of research project sponsored by a funding agency (NEDO). The color variation is due to the different size of aqueous CdTe-based QDs.

As a result, the first bulk glass containing dispersed emitting QDs was successfully fabricated, maintaining an initial PLQY of ~40%, as illustrated in Figure 4. Due to the release of methanol during the hydrolysis of APS, which acts as a poor solvent for QDs, the achievable dispersion concentration of QDs within the resultant silica matrix remained relatively low, typically in the order of 10^−5^ mol/L [55]. As the brightness of QDs is directly proportional to the dispersion concentration in appropriate conditions, higher concentrations are desirable for practical applications.

Similar results were reported more recently for hydrophobic CdSe QDs. Although TEOS was used in combination with other silane-coupling agents, its molar ratio was maintained below 10% [56]. Consequently, the resultant monoliths exhibit characteristics comparable to those of APS-based matrices, wherein the initial PLQY is relatively well preserved. Notably, the dispersion concentration of QDs achieved in this case is estimated to be approximately two orders of magnitude higher (5 wt%) than that reported in the earlier APS-based approach.

In the case of InP-based QDs, hydrophobic variants were first hydrophilized and subsequently incorporated into silica matrices synthesized from silicon compounds, exhibiting behavior similar to silane coupling agents. The PLQY was relatively well maintained, decreasing from an initial 52% to 27% upon encapsulation. However, insufficient development of the silica network is suggested by the absence of characteristic cracks which typically result from condensation and shrinkage in bulk materials. The stability was evaluated against PMMA [57]. In a subsequent effort, four-functional alkoxides (TMOS and TEOS) were employed for encapsulating the same hydrophobic QDs. In the case of TMOS, a high PLQY was reported, likely reflecting the properties of QDs embedded in a gel-like matrix [58].

### 3.2. Layer-by-Layer (LbL) Incorporation of QDs Using APS

**Figure 5:**
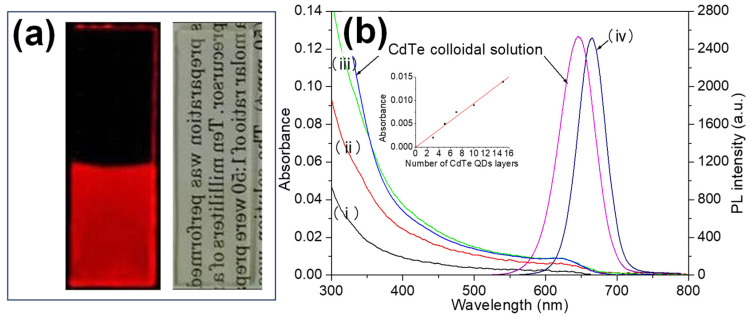
(**a**) PL image of the self-assembled films irradiated with 365 nm UV light prepared using APS and CdTe colloidal solution (left). A photograph of the sample taken under ambient room lighting (right). (**b**) PL and absorption spectra of the films. Spectra (i), (ii), and (iii) correspond to absorption measurements for samples with 3, 5, and 10 CdTe layers, respectively. Spectrum (iv) represents the PL response of the film with 10 CdTe layers. The inset in (**b**) illustrates the absorbance at the first absorption peak as a function of the number of dipping cycles. For comparison, the PL and absorption spectra of diluted QD colloidal solutions are also provided [59].

To enhance QD incorporation into silica matrices, a layer-by-layer (LbL) deposition method was employed using APS [59], building upon the previously discussed bulk encapsulation results. In this approach, a glass slide was sequentially dipped into APS solution, TGA-containing solution, and QD solution, forming transparent QD layers between the glass layers, as shown in Figure 5a. The inset of Figure 5b demonstrates a linear increase in absorbance with each successive dipping cycle, indicating progressive QD accumulation. Using the molar extinction coefficient of QDs [81], the concentration was estimated to be 10^−2^ mol/L, approaching the threshold of concentration quenching. At such high concentrations, the PL peak wavelength is red shifted with a narrowing of spectral width due to the re-absorption of PL. In this phenomenon, PL emitted from smaller QDs is reabsorbed by adjacent larger QDs, leading to selective emission primarily from the larger QDs. Consequently, the observed PL spectrum shifts toward the red end, with a reduction in full width at half maximum (FWHM). These observations confirm that APS facilitates effective QD incorporation, making it a preferred choice for both the bulk glass incorporation of hydrophobic QDs [82] and thin-film applications [83].

When the applied sol–gel process does not require stirring, such as the LbL process, the QDs do not suffer from dissolution according to the mechanism outlined in Equation (1). This allows for a significant increase in dispersion concentration while largely preserving the initial PLQY. However, glasses prepared using only three-functional alkoxides, such as APS, lack a robust glass network both for bulk and LbL. As a result, they exhibit insufficient structural integrity and eventually dissolve completely in hot water.

## 4. Incorporation of QDs into Sol–Gel-Derived Silica Nanoparticles

As research progressed, it became evident that silica nanoparticles with encapsulated QDs are particularly promising for both display and biological tagging applications. Among encapsulation methods, the step-by-step grafting of alkoxide molecules [84,85] was limited in effectiveness, since this method cannot be used to achieve thicknesses beyond 10 nm. However, excluding this approach, the sol–gel encapsulation method can generally be classified into two main categories: the reverse micelle method and the Stöber method. The reverse micelle method uses a water-in-oil emulsion where the alkoxide initially disperses in the continuous oil phase but gradually goes into the water droplet phase upon hydrolysis. In the Stöber method, the typical alkoxide TEOS reacts with an alkaline aqueous solution of abundant ethanol to form hydrolyzed TEOS, which later condenses onto colloidal QDs, resulting in the formation of nanosized silica particles with encapsulated QDs. As described above, the QDs are of two types: hydrophilic and hydrophobic. Therefore, the encapsulation methods result in four distinct types. The following sections detail each method separately.

### 4.1. Hydrophilic QDs Incorporated into Silica Nanoparticles via Reverse Micelle Method

**Figure 6:**
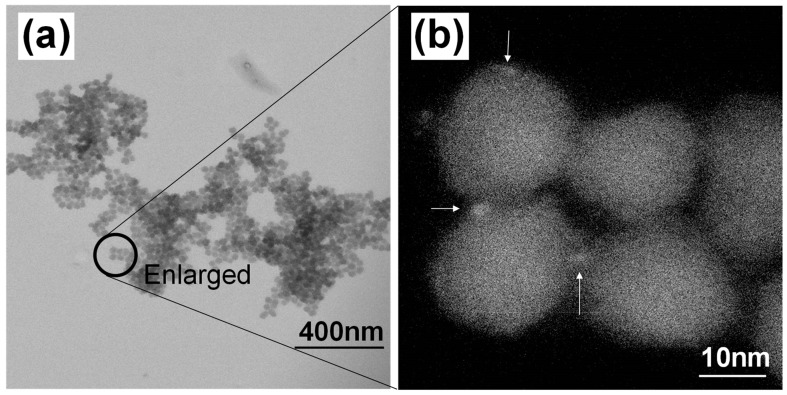
(**a**) Transmission electron microscope (TEM) image of silica spheres synthesized using a reverse micelle method with aqueous QDs in bright field mode. (**b**) High-angle annular dark field (HAADF) image of the region indicated by a circle in (**a**), highlighting locations where Cd is detected, as marked by the arrows [51].

For hydrophilic QDs, the reverse micelle method leverages water droplets within the water-in-oil microemulsion as their dispersion medium. Initially, hydrophobic TEOS molecules remain in the continuous oil phase but gradually migrate into the water droplet phase upon hydrolysis. Subsequently, hydrolyzed TEOS condenses in an alkaline environment, forming silica nanoparticles. The aqueous QDs (TGA-capped CdTe QDs) were initially expected to be incorporated into the silica particles by this protocol. However, the transmission electron microscope (TEM) image (Figure 6) revealed that the QDs were pushed away during the silica network formation, preventing effective encapsulation [51]. To overcome this challenge, a hydrophilic vitreous layer was introduced onto the QD surface prior to sol–gel processing, successfully enabling multi-QD incorporation into nanoscale silica particles. This refinement allowed red-emitting QDs to maintain a PLQY of 65% post-encapsulation [52].

In contrast, aqueous CdS QDs synthesized within reverse micelle droplets were successfully encapsulated using TEOS, without the need for additional surface modifications in advance [53]. This discrepancy appears to stem from CdS QDs being inherently stabilized in an alkaline environment (rich in hydroxyl ions) without requiring external ligands. However, PL properties for this system were not reported.

### 4.2. Hydrophilic QDs Incorporated into Silica Nanoparticles via the Stöber Method

In the early stages of research, hydrophilic QDs such as CdTe stabilized by short-chain thiols, or CdSe stabilized by sodium citrate, were incorporated into silica particles using sodium silicate. These silica particles containing multiple QDs were referred to as “Raisin Bun-type” silica composites. To further enhance their structure, the Stöber method was used to coat the silica spheres, increasing their size beyond 200 nm. However, at this stage, PLQY remained low, and the particles were primarily used to construct three-dimensional photonic crystals [60].

To improve encapsulation efficiency, a two-step process was developed for integrating multiple TGA-capped CdTe QDs into silica particles [61].

Step 1—QD Assembly Using MPS: Since thiol ligands exhibit the strongest binding energy to II-VI QDs, an aqueous CdTe QD solution was mixed under alkaline conditions with ethanol solution containing (3-mercaptopropyl)trimethoxysilane (MPS, a silane coupling agent) for the partial exchange of TGA ligands. A controlled amount of ethanol and MPS facilitated the formation of CdTe QD assemblies.

Step 2—Silica Layer Formation via the Stöber Method: A silica coating was developed on the assembled QDs using TEOS and aqueous ammonia via the Stöber method. The resulting silica nanoparticles were collected by centrifugation and re-dispersed in pure water.

#### 4.2.1. Nanoparticle Morphology and PL Properties

**Figure 7:**
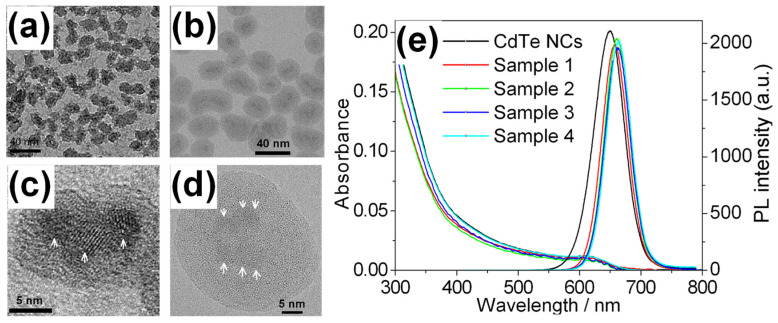
TEM images of luminescent silica nanoparticles. (**a**) Sample 2, (**b**) Sample 3, (**c**) and Sample 2 at a higher resolution, and (**d**) Sample 3 at a higher magnification. Three and six QDs (indicated by white arrows) are visible in (**c**) and (**d**), respectively. Well-developed lattice fringes are observed in CdTe QDs, confirming their structural integrity. (**e**) Absorption and PL spectra of luminescent silica nanoparticles, compared with those of initial CdTe QDs (nanocrystals, NCs). After encapsulation, PL peaks are red-shifted and a spectral narrowing is observed compared with original QDs [61].

The typical morphologies of silica nanoparticles prepared in Reference [61] are shown in Figure 7a–d. In the best-case scenario, PLQY was reduced slightly from its initial value (46%) to 40% upon encapsulation. Given the close packing of QDs within each nanoparticle, PL re-absorption occurs, similar to the behavior described in Section 3.2. This results in an overall red shift in the PL spectrum and a narrowed FWHM as shown in Figure 7e. By adjusting the MPS concentration in Step 1, the number of QDs per nanoparticle could be effectively controlled. Table 3 outlines the relationships between various parameters, including PL peak wavelength, PLQY, FWHM, nanoparticle size, and QD count.

#### 4.2.2. Hybrid Structure and Porous Properties

The silica nanoparticles exhibit a hybrid structure in the central part, primarily due to the combined presence of MPS and TEOS used in Step 1. The MPS concentration has a decisive role to control the number of QDs in the assembly. Higher MPS concentration results in fewer QDs in the central region after Step 2. This effect is quantitatively explained in the footnote of Table 3.

The porous nature of the silica nanoparticles thus prepared was evaluated by N_2_ adsorption–desorption isotherms. When the silica layer thickness exceeded ~10 nm, the shielding ability improved due to a pore transition from cylindrical to ink-bottle-shaped pores. These structural behaviors differ significantly from those observed in bulk silica systems [62], where drying-induced shrinkage plays a key role in pore formation. Based on the insights into porous characteristics, a shell thickness exceeding 10 nm was prepared via the Stöber method, as described in the following section using hydrophobic QDs. The excellent shielding effects are detailed in Section 5.4.

The potential for biomedical applications was demonstrated by successfully conjugating biotinylated immunoglobulin to the prepared silica nanoparticles with a shell thickness of ca. 10 nm [61]. Quite similar results were obtained using aqueous InP-based QDs as well [63]. These suggest promising future directions for bio-tagging technologies utilizing QD-incorporated silica nanoparticles.

### 4.3. Hydrophobic QDs Incorporated via Reverse Micelle Method

Following the successful synthesis of highly emitting CdSe-based QDs [13,78], several researchers attempted to incorporate them into silica particles [64,65]. The most comprehensive study on encapsulation mechanisms was conducted by Meijerink’s group in the Netherlands [66]. In their approach, CdSe-based QDs, typically octadecylamine-capped, were mixed with TEOS, surfactant, and aqueous ammonia in cyclohexane. This process rapidly exchanged the pristine alkyl-amine ligands, rendering the QDs hydrophilic at the expense of a significant PLQY reduction. The encapsulation process followed similar steps to those used for hydrophilic QDs, yet the final silica particles exhibited PLQY decay to below a few percent typically within one week.

A similar encapsulation approach was reported by another research group, involving oleic-acid coated CdSe-based QDs, instead of those coated with long-chain thiols. While the choice of the surfactant used in microemulsion system differed, the study similarly observed a time-dependent reduction in PLQY [67].

Single CdSe-based QDs were successfully encapsulated within individual silica particles, emphasizing surface silanization as a key factor in maintaining PL stability throughout the reverse micelle process [68]. For red-emitting QDs, the initial PLQY of 60% decreased to 35% post-encapsulation, while the PL spectral width (FWHM) narrowed from 28 nm to 24 nm. Impressively, the PLQY remained stable for months in aqueous conditions. Further investigations revealed that silanization conditions, particularly the molar ratio between QD and TEOS, play a crucial role in enhancing photostability [69].

### 4.4. Hydrophobic QDs Incorporated via the Stöber Method

An early study described the encapsulation of CdSe/ZnS QDs following ligand exchange with MPS. Researchers explored conditions for incorporating single QDs within individual silica particles [70], though PLQY values before and after encapsulation were not reported.

Around the same time, another study [71] demonstrated the formation of silica spheres incorporating CdS/ZnS QDs localized in the surface region. Initially, hydrophobic tri-n-octylphosphine oxide (TOPO) ligands on the QDs were replaced with two types of amino compounds, enhancing ethanol dispersibility. Similarly to the approach used for hydrophilic QDs, these vitreophilic QDs were then embedded within a silica layer (~100 nm thick) surrounding monodisperse silica microspheres (~550 nm in diameter). However, the resultant PLQY dropped from 38% to 13%. A limitation of this method is that the central silica core does not contribute to PL, reducing overall intensity.

Seemingly, the Stöber method appears to reduce the PLQY significantly because of the required ligand exchanges that facilitate the deposition of silica molecules onto the QDs. Additionally, lower alcohols commonly used to precipitate both hydrophilic and hydrophobic QDs often compromise their stability. Recognizing these challenges, the authors developed a new approach to integrate dozens of hydrophobic QDs into the central region of nanosized silica spheres while preserving the original PL properties. This approach is discussed in detail in the next section.

## 5. Silica Nanoparticles with Encapsulated Dozens of Hydrophobic CdSe-Based QDs

### 5.1. Preparation and Formation Mechanism

**Figure 8:**
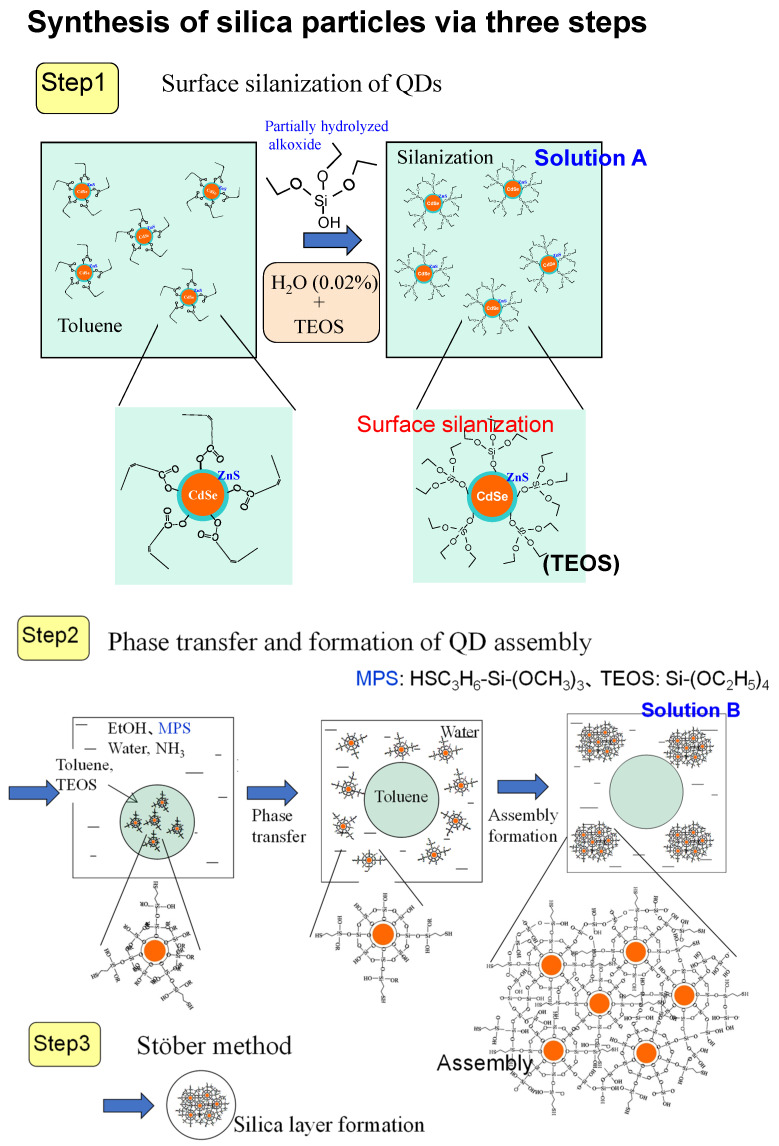
Schematic illustration of the process for assembling multiple CdSe/ZnS QDs into silica nanoparticles. The procedure consists of three key steps: (1) surface silanization, where QDs are modified to enhance compatibility with silica precursors; (2) phase transfer, facilitating QD transition from an organic to an aqueous phase; and (3) growth of the assembly, leading to encapsulation within silica matrices. Solution A contains silanized CdSe/ZnS QDs dispersed in toluene; Solution B consists of partially hydrolyzed MPS in ethanol, H_2_O, and ammonium hydroxide (NH_4_OH), essential for controlled nanoparticle formation [72].

As discussed earlier [61], a silica layer thicker than 10 nm is crucial for effective shielding against the environment. However, encapsulating a single QD per silica particle results in an overall particle size of nearly 30 nm, which limits the high dispersion concentration required for applications. Consequently, smaller silica particles (several tens of nanometers in size) with dozens of embedded QDs have proven to be more suitable. Hydrophobic QDs synthesized at approximately 300 °C exhibit high PLQY and narrow FWHM, making them preferable for phosphor applications rather than hydrophilic QDs (as described in Section 4.2). Nonetheless, the synthetic method utilizing MPS as a size regulator remains a valuable approach. The encapsulation of CdSe-based QDs consists of three steps, as illustrated in Figure 8 [72,73].

Step 1—Surface Silanization: TEOS is added to a toluene solution containing hydrophobic QDs capped with oleic acid. Since analytical-grade toluene contains a small amount of water (~0.02 wt% [43]), a single alkoxy group among the four in TEOS gradually hydrolyzes, forming (CH_3_CH_2_O)_3_-Si-OH, which replaces the pristine ligand molecule, a process termed surface silanization. Due to the slow ligand replacement, the partially hydrolyzed TEOS forms a well-ordered arrangement on the QD surface, ensuring effective passivation without changing the PLQY and FWHM of the initial QD. This step enhances the QD stability against lower-alcohol exposure.

Step 2—Formation of QD Assembly (Seed): The silanized QDs in toluene are mixed with hydrolyzed MPS in an aqueous ammonia and ethanol solution. Since the QDs in toluene are efficiently exposed to the aqueous phase because of abundant ethanol, they are hydrolyzed more, become hydrophilic, and finally are transferred into the aqueous phase. In this aqueous phase, QDs are condensed rapidly to form seed structures. Since MPS hydrolyzes slower than TEOS, MPS acts as a size regulator, similar to its role in hydrophilic QD encapsulation. A higher concentration of MPS results in smaller QD assemblies, with a lower QD count per particle.

Step 3—Silica Layer Formation via the Stöber Method: Following purification of the solution in Step 2, the Stöber method is applied to deposit a silica layer around the assembled QD structures, completing the encapsulation process. 

### 5.2. Structural and Optical Properties

**Figure 9:**
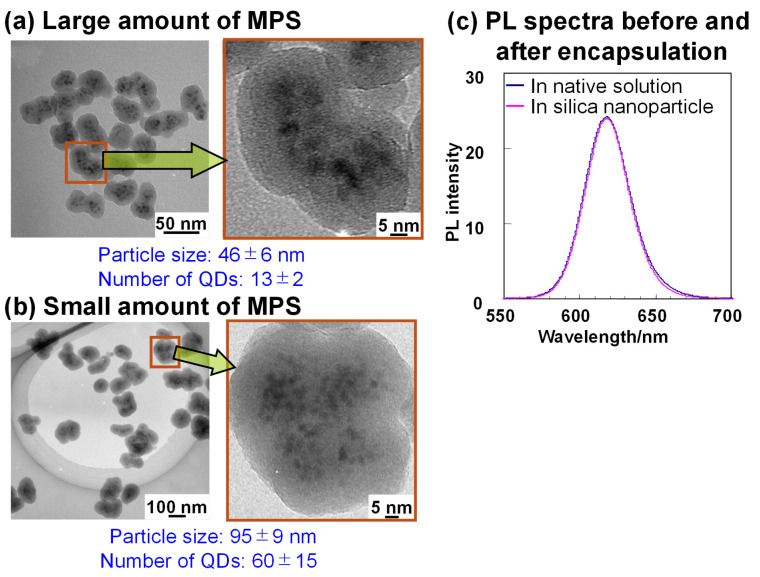
TEM images of luminescent silica nanoparticles displaying (**a**) particle 1 and (**b**) particle 2. The high-resolution images on the right provide a detailed visualization of the distribution of QDs in nanoparticles [72]. (**c**) Comparison of PL spectra of the colloidal solution and nanoparticle shown in (**a**).

The morphology and PL spectra of the prepared silica nanoparticles are presented in Figure 9. The assembly size was successfully controlled by adjusting MPS concentration, as evidenced by the differences between Figure 9a and b. Additionally, Figure 9c confirms that PL spectral shape remains intact after the encapsulation. A three-dimensional imaging study [72] further reveals QD distribution within silica nanoparticles, distinctly visible as white dots. Initially, the synthesis focused solely on QDs emitting at ~620 nm, as shown in Figure 9c. To extend applicability, the preparation method was optimized for various PL wavelengths. It was determined that the QD concentration in Step 1, relative to the TEOS amount, must be optimized to achieve the maximum PLQY in silica nanoparticles. This optimization is critical because silanization efficiency in Step 1 depends on the total QD surface area in solution.

**Figure 10:**
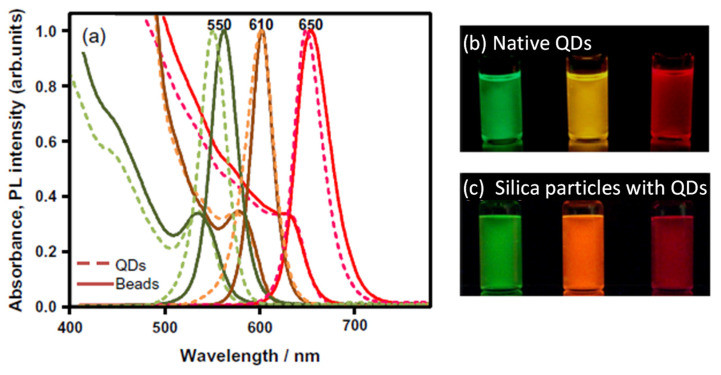
(**a**) Normalized absorption and PL spectra of CdSe/CdZnS QDs in toluene solution and in silica nanoparticles (beads). Numbers at the top of the graph indicate the PL peak wavelengths of QDs in toluene. (**b**) PL image of as-prepared QDs in toluene solution, and (**c**) PL image of QDs incorporated in silica particles, both excited by a UV lamp at 365 nm, illustrating emission characteristics before and after encapsulation [74].

The resultant PL spectra and PL images emitting initially at 550, 610, and 650 nm before and after the encapsulation are shown in Figure 10a. Notably, when smaller QDs are encapsulated, PL red shifting becomes more pronounced due to the additional influence of the quantum confinement effect associated with the smaller size of the QDs [74], despite similar reabsorption phenomena being observed and previously described in Figure 5b and Figure 7e.

### 5.3. PL Properties at the Single-Particle Level

**Figure 11:**
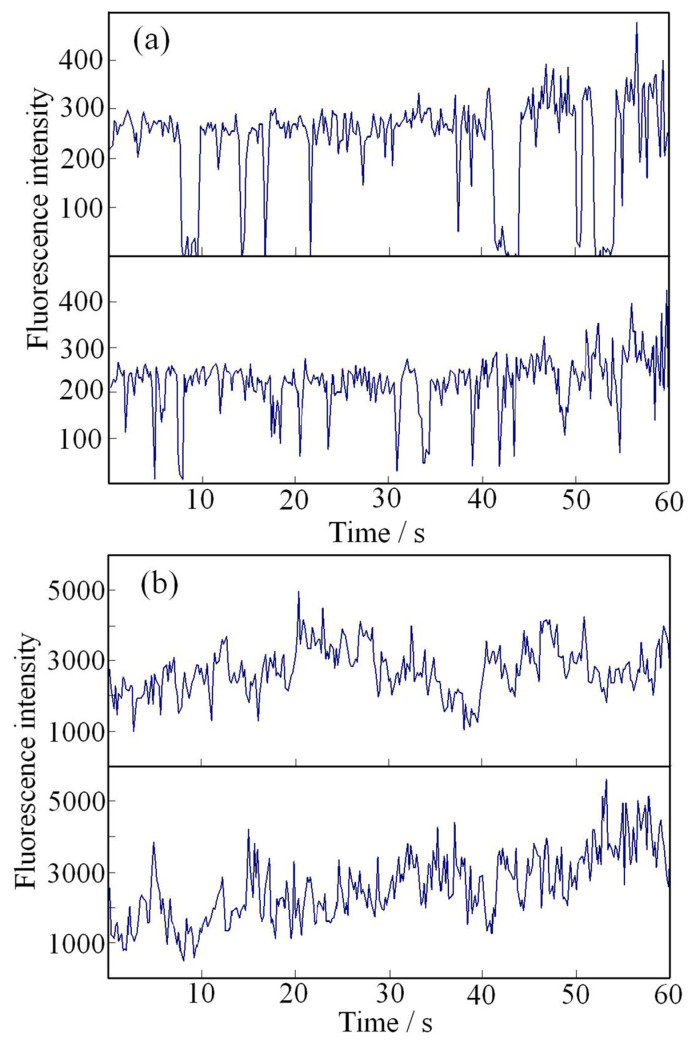
PL intensity trajectories of single QDs and silica particles, recorded with a temporal resolution of 200 ms over a duration of 60 s: (**a**) PL intensity of two random individual CdSe/ZnS QDs, exhibiting pronounced blinking behavior. (**b**) PL intensity of two random individual luminescent silica particles, demonstrating a non-blinking nature, with only minor fluctuations in intensity. Note that the average PL intensity of silica particles (**b**) was approximately 15 times higher than that of individual QDs (**a**) under comparable conditions, indicating enhanced emission in the silica matrix [72].

Evaluations were conducted on the prepared silica nanoparticles to analyze their PL behavior at the single-particle level. Using a standard single-particle detection method, the PL intensity of two random pristine QDs exhibited distinct blinking, as illustrated in Figure 11a. However, in contrast, the PL intensity of two random silica nanoparticles displayed only fluctuations rather than blinking. Here, it is reasonable to consider that each individual QD within a single silica particle does not interact with others because the overall ensemble PL spectrum in Figure 9c remains unchanged after encapsulation. This means each QD in the particle exhibits the same blinking nature as pristine QDs before encapsulation. The PL originating from the ensemble of approximately 20 QDs per silica nanoparticle results in fluctuations within the measured temporal resolution of 200 ms. The apparent non-blinking behavior observed in Figure 11b is attributed to this exposure window, which masks the detail of individual blinking events. The variations likely reflect the cumulative blinking dynamics of the constituent QDs within the same nanoparticle. A crucial observation is that the PL intensity (vertical axis) from the silica nanoparticles in Figure 11 is approximately 15 times stronger than that of a single QD under similar conditions. The original fluorescence microscope recordings used for Figure 11, provide further evidence of this behavior for both pristine QDs and silica nanoparticles [72]. Notably, after continuous irradiation for 30 min, the silica nanoparticles exhibited significantly higher photostability compared to their pristine QD counterpart.

The observed substantially non-blinking behavior, coupled with strong PL from individual silica-encapsulated QD particles, is particularly advantageous for in vivo imaging applications [86]. This capability enables the simultaneous tracking of multiple biomolecular targets with enhanced spatial resolution and temporal resolution. Furthermore, silica surfaces offer well-established platforms for bioconjugation [87], facilitating targeted delivery and functionalization. Togher with the substantial suppression of heavy metal elusion discussed in the following subsection, these silica-encapsulated QDs represent highly promising fluorophores for biological applications.

As outlined in the Introduction, several comprehensive review articles on biological applications of colloidal emitting QDs [4,5,6] have been published, following their initial development [13,78]. The most recent review [6] focuses on the pharmacokinetics and biodistribution of infrared-emitting QDs in murine models, demonstrating that surface modification and particle size critically influence distribution and excretion pathways. The silica-coating methodology discussed in this review is expected to significantly contribute to advancements in this promising research domain. Nonetheless, it is important to acknowledge that the extensive lead time and substantial cost associated with validation and regulatory approval continue to pose challenges for transitioning such technologies into clinical use for humans.

### 5.4. Elusion of Heavy Metal Ions and Cytotoxicity of Silica Nanoparticles

To assess the shielding performance of silica layers other than the photostability of PL intensity, two additional measurements were conducted to study the elusion of heavy metal ions (Cd^2+^) into buffer solutions and cytotoxicity testing in live cell culture mediums [75].

For the elusion analysis, two additional silica nanoparticles were synthesized by modifying the Stöber method in Step 3. In Table 4, B represents the silica nanoparticle described in the previous Section 5.1, while C was prepared by performing the Stöber method at 40 °C instead of room temperature. To enhance biocompatibility for biological applications, the silica surface initially capped with hydroxyl (-OH) groups was modified with carboxyl (-COOH) groups for bioconjugation. This was achieved using carboxyethylsilanetriol sodium salt (CES) during the Stöber process [72], resulting in silica nanoparticle D. Additionally, a commercial polymer-coated aqueous QD (A10200, surface-modified with COOH, Thermo Fisher Scientific Inc., Waltham, MA, USA) was used as a control and labeled A.

Stock solutions of silica nanoparticles and commercial polymer-coated QDs listed in Table 4 were precipitated and redispersed in HEPES buffer solution (4-(2-hydroxyethyl)-1-piperazineethanesulfonic acid) at concentrations ranging from 20 to 50 nM. After 15 h, the solution was filtered through a centrifugal concentration filter. The concentration of Cd^2+^ in the obtained filtrate (in the ppb range) was quantified using inductively coupled plasma mass spectrometry (ICP-MS). Table 4 presents the normalized Cd^2+^ concentrations, adjusted to a standard 10 nM QD concentration, using A as the reference. The schematic diagrams on the right-hand side of the Table illustrate the morphology of QDs and silica matrix network development. As observed, silica matrices demonstrate superior shielding performance compared to polymer coatings, effectively reducing heavy metal ion elusion. Additionally, variations in Stöber method conditions significantly impact silica network formation, highlighting the importance of synthesis optimization. These insights contribute to understanding discrepancies in reported cytotoxicity data for silica shells following meta-analysis [88].

**Figure 12:**
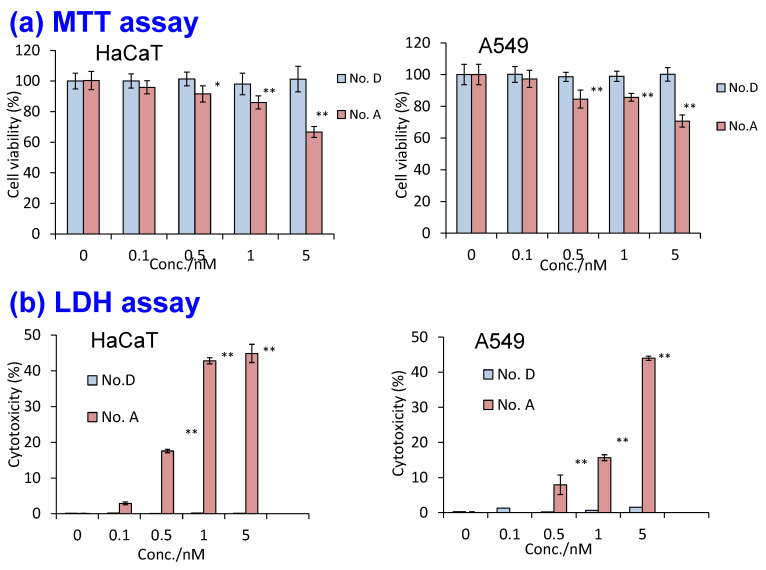
(**a**) MTT assay results for two human cell lines, HaCaT and A549, assessing cell viability based on mitochondrial activity. (**b**) LDH assay results for the same two cell lines, evaluating cell membrane damage. In this study, both mitochondrial function and membrane integrity were significantly affected only in cells exposed to polymer-coated QDs, whereas silica-encapsulated QDs demonstrated improved biocompatibility for both cell types [75]. Nos. A and D in the figure correspond to the samples A and D respectively shown in Table 4. All values are mean ± SD, * *p* < 0.05 vs. control, ** *p* < 0.01 vs. control. *p* < 0.05 is considered to indicate a statistically significant difference.

Cytotoxicity assessments complemented the heavy metal elusion study. The best silica nanoparticle (D) with a COOH-modified surface was compared to the control nanoparticle (A), which was also COOH-modified, using two widely recognized assays: MTT [3-(4,5-Dimethyl-2-thiazolyl)-2,5-diphenyltetrazolium Bromide] assay, which evaluates cell viability, and LDH (Lactate Dehydrogenase) assay, which assesses cell membrane damage. Two widely studied human cell lines, lung carcinoma A549 cells and keratinocyte HaCaT cells, were used for the evaluation. Figure 12 demonstrates that D exhibited no cytotoxicity, whereas A induced significant toxicity in both cell types, as evidenced by MTT and LDH assay results.

These cytotoxicity findings align with the heavy metal elusion trends shown in Table 4, reinforcing the correlation between silica shielding efficiency and biocompatibility. Moreover, silica layers synthesized via the Stöber method (Step 3) exhibit variations in shielding performance, suggesting that further optimization could be beneficial in developing robust silica matrices comparable to those widely utilized in semiconductor technology.

**Figure 13:**
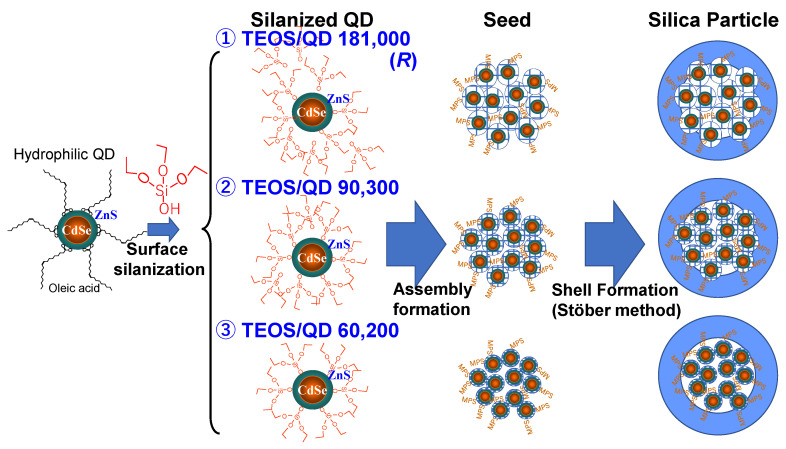
Reaction scheme illustrating the formation of three types (①–③) of silica particles, starting from hydrophobic QDs through surface silanization and assembly (seed) formation to shell (silica) formation. The initial ratio (*R*) of TEOS to QDs significantly influences the degree of condensation within the silica-glass network around the QDs in the particles. This dictates the difference in QD density and the photostability of silica particles [76].

**Figure 14:**
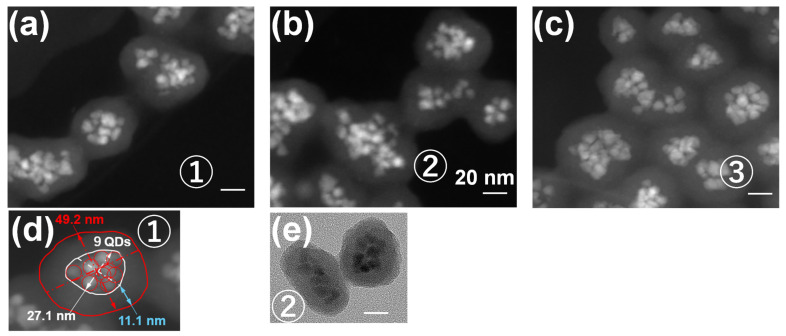
(**a**–**c**) HAADF images of three different types (①–③) of silica particles. The white scale bars in the images correspond to 20 nm. (**d**) Evaluation of the number of QDs present in type ① silica particles, including data on assembly (seed) size, overall particle dimensions, and shell thickness. The small red circles represent the outline of the QDs whereas the large red circle represents the outline of the entire nanoparticle. In addition, the white circle represents the outline of the seed. The particle size of nanoparticles and the thickness of silica shell are also shown. (**e**) Typical TEM image of type ② silica particles, corresponding to the HAADF image in (**b**) [76].

Expanding on Step 3, further investigations were conducted to assess how the preparation method, specifically the surface silanization conditions in Step 1, influences photostability [76]. Three types of nanoparticles, labeled ① to ③, were synthesized by varying only the initial ratio *R* of TEOS to QD, as shown in Figure 13, which directly impacted the QD density within the assembly and consequently the photostability of the silica particles. Morphological assessments were conducted using TEM and high-angle annular dark field scanning TEM (HAADF-STEM) (Figure 14). These images enabled precise quantification of QD count, assembly size, and silica shell thickness across all three nanoparticle types.

**Figure 15:**
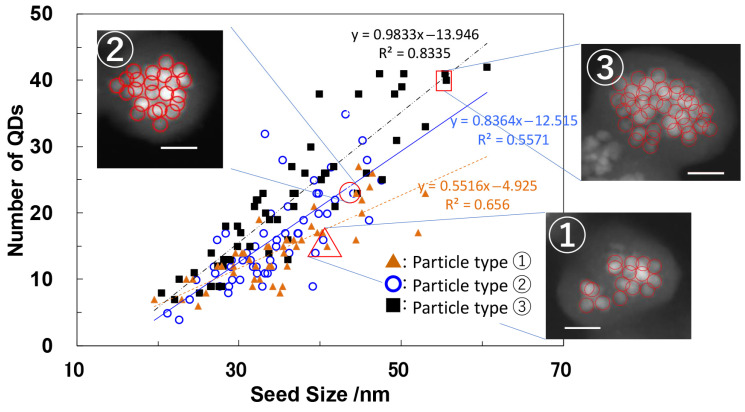
Relationship between the assembly (seed) size and the number of QDs for three types (①–③) of particles. The red circles in the images indicate the positions of QDs. The results of linear fitting are shown with corresponding equations and correlation coefficients. Three typical HAADF images used for counting the number of QDs in the particles are shown, with the data points marked by triangles, circles, and squares corresponding to types ①, ②, and ③, respectively. The white scale bars correspond to 20 nm [76].

**Figure 16:**
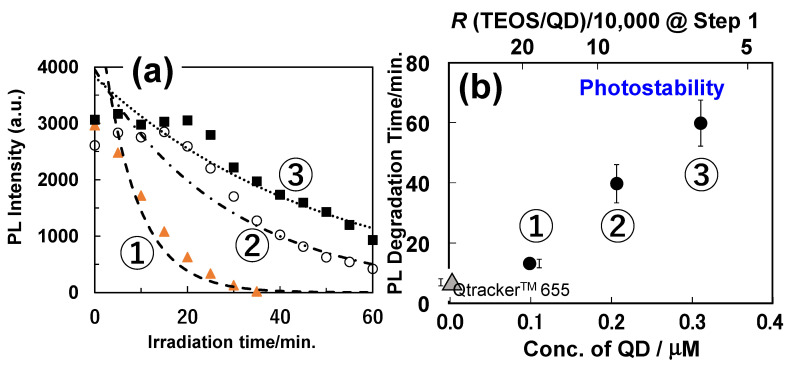
(**a**) PL intensity for three types (①–③) of nanoparticles against the irradiation time. Each data point was fitted using a single exponential decay curve. (**b**) PL degradation time with error bars for three types of nanoparticles. The result of commercial aqueous QD (Qtracker) is plotted on the vertical axis of the figure as a triangle for comparison [76].

Figure 15 illustrates the correlation between assembly size and QD number, with corresponding linear fitting equations. Among the three samples, nanoparticle ③ exhibited the slope, indicating the highest packing density of QDs, which resulted from its unique silanization condition. To assess photostability, single-particle detection was applied to more than 20 individual nanoparticles from ①, ②, and ③. Figure 16a depicts the overall PL intensity decay over irradiation time for all three samples. A commercial polymer-coated aqueous CdSe-based QD (Qtracker™ 655, Thermo Fisher Scientific Inc. Massachusetts, USA), known for its exceptional durability, was selected as a control due to its emission wavelength being nearly identical to that of the silica nanoparticles. Figure 16b plots PL degradation time with error margins derived from Figure 16a, plotted against *R* (TEOS/QD ratio) and QD concentration during Step 1. The same Qtracker™ 655 data is displayed as a filled triangle on the vertical axis. These evaluations revealed that nanoparticle ③ exhibited up to an eight-fold increase in photostability compared to polymer-coated counterparts, achieved by optimizing the molar ratio *R* of alkoxide to QDs during silanization.

The developed silica network formed in Step 1 near the QD surface remained structurally intact in Step 2, where hydrolyzed MPS was introduced to regulate assembly size. Ultimately, controlled silanization in Step 1 proved to be as significant as Step 3 in improving the photostability of silica nanoparticles.

## 6. Recent Progress and Applications

### 6.1. Adapting Earlier Approaches for Silica Nanoparticle Encapsulation with InP-Based QDs

As outlined in the background section, InP-based QDs stand out as the only environmentally benign binary system capable of emitting in the visible range. With this objective, the previously established silica nanoparticle encapsulation method, which was originally developed for hydrophobic CdSe-based QDs, was adapted for InP-based QDs. However, following this method, a significant decrease in PLQY was unexpectedly observed. To understand this discrepancy, three variations of InP-based QDs were synthesized, specifically InP/(ZnSe)*_n_*/ZnS, with *n* = 4, 6, and 8 monolayers (MLs), each emitting at approximately 550 nm. These QDs were subsequently encapsulated within silica nanoparticles to investigate the influence of shell thickness and surface interactions on PLQY retention.

**Figure 17:**
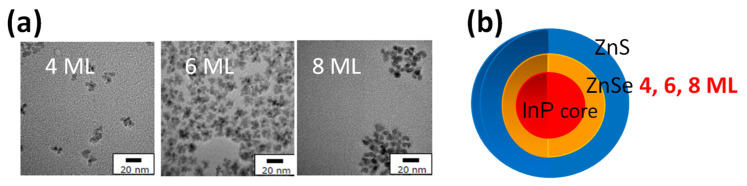
(**a**) TEM images of InP-based QDs synthesized with 4, 6, and 8 monolayers (ML) of ZnSe using the same core structure. (**b**) Schematic representation of the three types of InP/(ZnSe)*_n_*/ZnS QDs (*n* = 4, 6, 8) used in this study [77].

**Figure 18:**
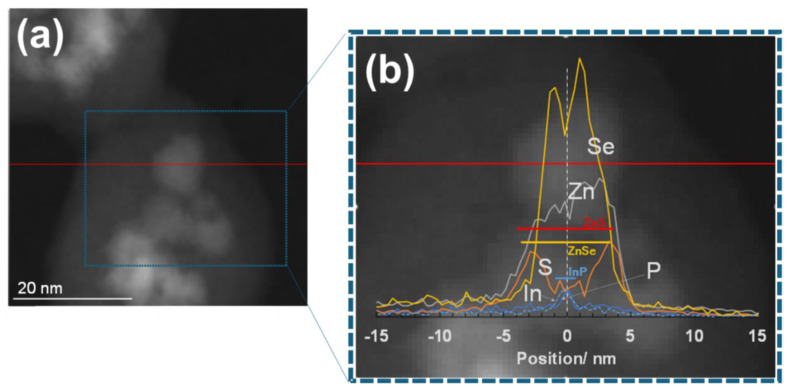
(**a**) Enlarged HAADF-STEM image of silica particles with encapsulated InP-based QDs (8 ML). The EDX analysis was performed along the red line across a single isolated QD. (**b**) EDX results are overlaid on the HAADF-STEM image. The horizontal bars (InP, ZnSe, ZnS) are drawn, corresponding to the preparation conditions used for QD synthesis and encapsulation [77]. The white vertical dashed line in (**b**) indicates the center line of the QD to be analyzed.

The TEM images and a schematic diagram of the prepared QDs are shown in Figure 17. Additionally, a HAADF-STEM image of one silica nanoparticle with embedded QDs (8 ML) is shown in Figure 18a. A single isolated QD in the silica matrix was selected for energy dispersive X-ray spectroscopy (EDX) analysis along the red horizontal line in Figure 18a, confirming in Figure 18b that the QD maintains its InP/ZnSe/ZnS structure. The findings suggest that QDs with a thicker intermediate ZnSe layer do not deteriorate significantly when encapsulated within silica nanoparticles.

**Figure 19:**
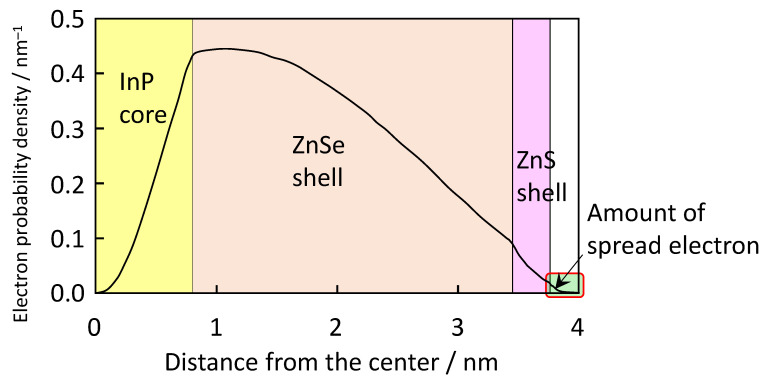
Radial probabilities for the presence of exciton electrons in QDs with 8 ML of ZnSe (InP/(ZnSe)_8_/ZnS). The amount of spread electron (electron outside the QD) is shown on the right-hand side, illustrating the degree of confinement within the QD [77].

**Figure 20:**
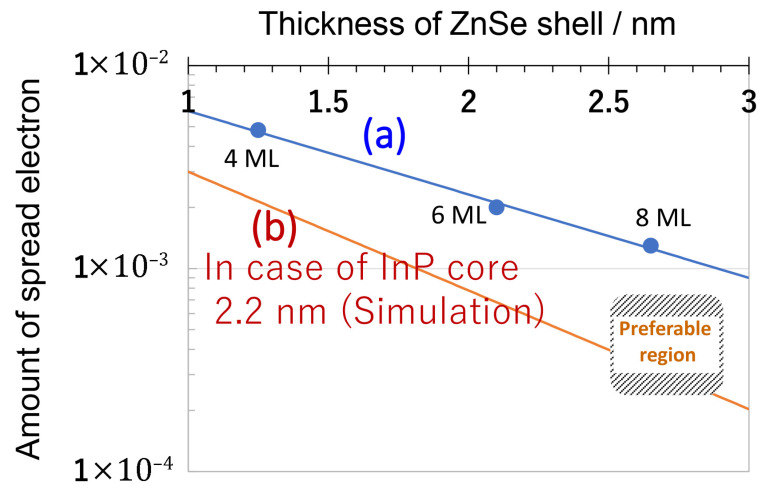
Relationship between the thickness of the ZnSe layer (InP/(ZnSe)_4,6,8_/(ZnS)) and the amount of spread electron outside the QDs. (**a**) Three QDs investigated in this study. (**b**) Simulation for an InP core diameter of 2.2 nm as a function of ZnSe-layer thickness. The preferable structure region of the QD for silica encapsulation is indicated by diagonal lines [77].

Since the nanoparticle encapsulation process involves initial hydrophobic surface ligand exchange with partially hydrolyzed TEOS molecules, the QD surface conditions significantly influence the resulting PL properties. To further elucidate the underlying photophysical behavior, quantum mechanical simulations were carried out to calculate the electron distribution within the exciton for these three InP-based QDs. One example of a QD with an 8 ML intermediate ZnSe layer is shown in Figure 19. As depicted in the right-hand region of Figure 19, the electron spreads out from the QD structure into the surrounding environment. The relationship between ZnSe-shell thickness and electron spread is depicted by a line (a) in Figure 20, revealing a linear trend when plotted on a logarithmic scale. It is important to note that CdSe-based QDs, discussed in the previous section, typically feature shell thicknesses between 0.5 and 1.5 nm. Compared to CdSe-based QDs, InP-based QDs tend to exhibit greater electron dispersion due to their lighter effective electron mass and lower barrier height from the InP core to ZnSe/ZnS shells. As the ZnSe layer thickness increases, the amount of spread electron reduces, thereby better preserving the PLQY after encapsulation. On the other hand, the degree of electron spread outside the QD serves as a key factor in PLQY reduction for InP-based QDs. The simulations further indicate that the preferable structure for QDs includes an InP-core size greater than 2.2 nm with a ZnSe shell thickness of 2.5 nm or more, resulting in an electron spread outside the QD in the order of 10^−5^ or less, as shown in the figure [77].

### 6.2. Phosphors for Display Technologies and Mass Production Strategies

**Figure 21:**
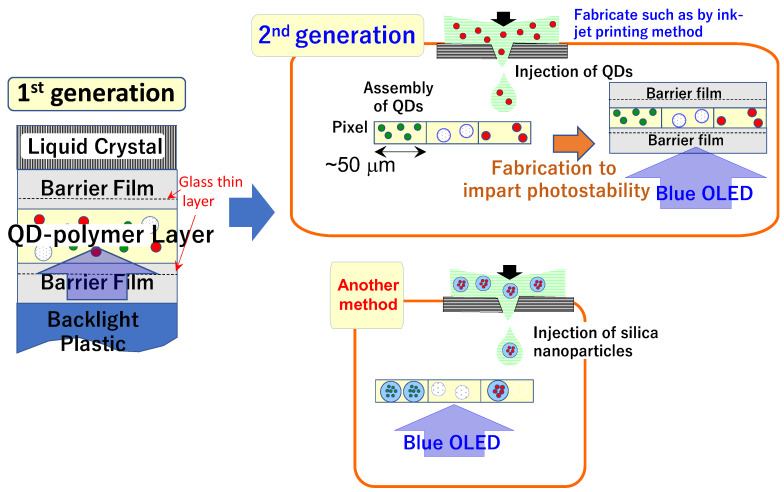
Schematic representation of display panel structures. In the first generation, the QDs are dispersed within a polymer layer and sandwiched between barrier films, including glass films. In the next generation, each pixel is formed by QD assembly. To enhance long-term stability, robust silica particles are proposed as a substitute for free QDs, reducing the need for additional stability measures during operation.

Figure 21 presents a schematic representation of the display panels currently available as commercial products. In the first-generation models, QDs were dispersed within a polymer layer (combining both green- and red-emitting QDs together with scattering materials such as titania) and sandwiched between barrier films, including glass films [2]. In subsequent generations, individual pixels were formed by separately assembled green- and red-emitting QDs, eliminating the need for liquid crystal while still requiring barrier films for stability. The robust silica particles are expected to replace free QDs, particularly those surrounded by polymers, thereby enhancing durability without necessitating additional stability measures during usage, as depicted at the bottom of Figure 21.

One of the main challenges in developing silica-encapsulated QDs was the lengthy preparation protocol, spanning steps 1 through 3 and requiring centrifugation at 50,000× *g* for separation. However, recent refinements have allowed the protocol to be streamlined into a single-pot process, reducing the necessary centrifuge speed to nearly half, significantly improving scalability for mass production. These silica particles are now being explored for broader applications beyond displays, offering a user-friendly phosphor with narrow spectral width, high brightness, and robust durability. Future investigations will focus on infrared emission using QDs composed of alternative heavy metals, particularly for biomedical applications [6] and infrared detection [89].

To drive commercialization, a start-up company [90] has been launched to leverage the findings presented in this article, particularly in optimizing phosphor materials for display technologies and expanding into infrared-emitting QDs. This effort entails detailed technical refinement and strategic negotiations to facilitate efficient manufacturing processes.

## 7. Concluding Remarks

In previous studies, the coating materials for QDs were compared, specifically polymer and silica [15]. Currently, more than five million QD displays are manufactured annually worldwide [3] using polymer coatings. However, due to their limited robustness, barrier films remain necessary. Ongoing research has actively addressed this challenge by fabricating silica nanoparticles encapsulating both hydrophobic and hydrophilic QDs. This dual encapsulation approach allows for broader material compatibility and facilitates comprehensive evaluation of photostability, dispersion uniformity, and biocompatibility across varied surface chemistries. The uses of silane coupling agents (initially APS and later MPS) have proved essential in controlling the overall morphology and maintaining the unique spectral properties of QDs.

Among these developments, silica nanoparticles with encapsulated dozens of hydrophobic CdSe-based QDs, synthesized through a three-step process using MPS (No. F in Table 2), demonstrated superior shielding effects. This was validated through PL measurements at the single-particle level, assessments of heavy metal ion (Cd^2+^) elusion, and cytotoxicity tests. The preparation steps, particularly steps 1 and 3, were found to significantly affect the effectiveness of the shielding. Furthermore, when applying this encapsulation method to hydrophobic InP-based QDs (InP/ZnSe/ZnS), a considerable decrease in PLQY was observed. Experimental comparisons and quantum mechanical calculations revealed that maintaining a thick intermediate ZnSe layer (greater than 2.5 nm) was necessary to mitigate this deterioration. This requirement arises due to the lower effective mass of electrons and the shallow barrier height between the core and shell, distinguishing InP-based QDs from their CdSe-based counterparts.

Since emitting QDs cannot withstand prolonged exposure to high temperatures, sol–gel preparations were conducted at moderate temperatures, typically in the range of one hundred degrees Celsius. The findings from this research indicate that with careful optimization, silica layers can achieve an unprecedented level of shielding performance even at relatively low temperatures, presenting promising opportunities for future applications.

## Figures and Tables

**Table 1:** Properties of transparent matrices for encapsulation of emitting QDs ^(a)^.

		Property	Shielding Ability (Photostability)	PLQY	Main Producer
		
Matrix					
Organic	**Amorphous**	Polymer	－	＋	General
**Inorganic**		**Silica** ^(b)^	＋	＋/－	The authors
	Crystal	Zeolite	＋	－	Korean group [27]
		Alumina	＋	－	Crystalplex Inc., USA [28], Related papers [29,30]

^(a)^ A plus sign (+) in the table indicates a positive or promising outcome, while a minus sign (−) denotes a negative or unfavorable result. ^(b)^ An inorganic and amorphous matrix shown in blue is preferable to encapsulate the crystalline QDs with preventing the intrusion of oxygen. We therefore have selected silica as shown in a thin yellow background.

**Table 3:** Properties of luminescent nanoparticles [61] ^(a)^.

Sample	PL Peak /nm	PLQY (%)	FWHM /nm	Mean Size /nm ^(b)^	Average QDs No. in Each Nanoparticle ^(b)^
1	658.4	34	49.2	12.5 ± 1.7	1.6 ± 0.7
2	660.8	40	48.0	15.9 ± 1.6	2.9 ± 0.9
3	662.6	31	48.4	29.1 ± 3.8	4.0 ± 1.6
4	663.8	32	47.2	35.9 ± 4.4	5.7 ± 2.3
CdTe ^(a)^	650.2	46	58.0	3.9 ± 0.2	—

^(a)^ Properties of initial CdTe QDs are shown for comparison. The molar ratios of MPS to QDs for Samples 1, 2, 3, and 4 during preparation were 176, 117, 57, and 29, respectively. ^(b)^ Estimated by TEM observation.

**Table 4:** Concentration of dissolved Cd^2+^ from various QDs dispersed in HEPES solution.

No.	Sample	Concentration Ratio of Dissolved Cd^2+^ in HEPES Solution ^(a)^	
**A**	Polymer-coated QDs (-COOH surface)	1	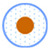
**B**	Silica nanoparticle with QDs (-OH surface, prepared at room temperature)	0.26	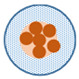
**C**	Silica nanoparticle with QDs (-OH surface, prepared at 40 °C)	0.06	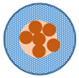
**D**	Silica nanoparticle with QDs (-COOH surface, prepared at room temperature)	0.009	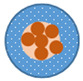

^(a)^ The values are normalized using the measured concentration listed in Table 1 of Reference [75]. A schematic illustration depicting the development of each silica network is shown on the right-hand side.
